# The Impact of Maternal Eating Disorders on Dietary Intake and Eating Patterns during Pregnancy: A Systematic Review

**DOI:** 10.3390/nu11040840

**Published:** 2019-04-13

**Authors:** Annica F. Dörsam, Hubert Preißl, Nadia Micali, Sophia B. Lörcher, Stephan Zipfel, Katrin E. Giel

**Affiliations:** 1 Department of Psychosomatic Medicine and Psychotherapy, Medical University Hospital Tübingen, 72076 Tübingen, Germany; Sophia.Loercher@med.uni-tuebingen.de (S.B.L.); Stephan.Zipfel@med.uni-tuebingen.de (S.Z.); Katrin.Giel@med.uni-tuebingen.de (K.E.G.); 2 Institute for Diabetes Research and Metabolic Diseases of the Helmholtz Center Munich at the University of Tübingen; fMEG Center; German Center for Diabetes Research (DZD), 72076 Tübingen, Germany; Hubert.Preissl@uni-tuebingen.de; 3 Department of Psychiatry, Faculty of Medicine, University of Geneva, 1205 Geneva, Switzerland; nadia.micali@hcuge.ch

**Keywords:** anorexia nervosa, bulimia nervosa, binge eating disorder, diet, eating behavior, eating disorders, nutrition, pregnancy, purging

## Abstract

Maternal nutrition in pregnancy has a key influence on optimum fetal health. Eating disorders (EDs) during pregnancy may have detrimental effects on fetal growth and the child’s early development. There is limited knowledge concerning the eating behavior, dietary intake and derived nutritional biomarkers as well as the nutrient supplementation in women with EDs during pregnancy. We performed a systematic review according to the PRISMA statement to synthesize current evidence in this field. Of *N* = 1203 hits, 13 full-texts were included in the qualitative synthesis. While women with current Binge Eating Disorder (BED) showed higher energy and fat intakes during pregnancy, women with a lifetime Anorexia Nervosa (AN), Bulimia Nervosa (BN) or both (AN + BN) had similar patterns of nutrient intake and dietary supplement use as healthy women. There is evidence, that women with a history of EDs have a sufficient diet quality and are more likely to be vegetarian. Dieting and bingeing improved substantially with pregnancy. The highlighted differences in the consumption of coffee/caffeine and artificially sweetened beverages as well as the elevated prevalence of iron deficiency anemia in women with a past or active ED during pregnancy might have an important impact on fetal development.

## 1. Introduction

In pregnancy, a lifestyle characterized by regular exercise and a balanced and diverse diet is a significant determinant of the course of pregnancy, the child’s development, and the short- and long-term health of the mother and child [1]. In relation to the slight increase in energy intake in the last months of pregnancy, the demand for some vitamins and minerals (including trace elements) increases significantly more [1], usually from the 4th month of pregnancy on [2]. For the nutrients folate and iodine, a markedly increased intake is recommended from the beginning of pregnancy or, ideally, before conception [2]. There is substantial interest in nutrition during pregnancy, due to the extensive research in the area of early nutrition programming [3], arisen from the initial work by Barker (‘Fetal Origins of Adult Disease’; FOAD) [4]. Barker’s initial concept was later modified to the ‘Developmental Origins of Health and Disease’ (DOHaD) [5], which postulates that exposure to certain environmental influences during critical periods of development (e.g., intrauterine deficiency or oversupply of nutrients) and growth may have a significant impact on the development of non-communicable diseases (NCDs), such as obesity, diabetes mellitus, cardiovascular and mental disorders, as well as cancer in later life [5,6,7].

Given that eating disorders (EDs) are characterized by dysfunctional eating behaviors (e.g., caloric restriction, purging behavior), which may result in specific maternal macro- and micronutrient deficiencies, one would predict negative consequences of maternal EDs on the health, growth, and development of the fetus and the newborn infant. Research suggests that up to 7.5% of pregnant women are affected by an ED [8]. EDs include Anorexia Nervosa (AN), Bulimia Nervosa (BN), Binge Eating Disorder (BED) and ‘Other Specified Feeding or Eating Disorders’ (OSFED) [9]. AN is characterized by an excessive restriction of energy intake, which leads to severe weight loss with a pathological fear of weight gain and a distorted body image [9]. BN is defined by regular episodes of binge eating followed by inappropriate compensatory behaviors such as self-induced vomiting, abuse of laxatives, fasting or excessive exercise to avoid weight gain [9]. BED is associated with recurring episodes of eating significantly more food in a short period of time than most people would eat under comparable conditions, accompanied by feelings of lack of control, guilt, embarrassment or disgust [9].

There is increasing evidence from large cohort studies and register data showing that maternal disordered eating behavior and dysregulated body weight have detrimental effects on the course of pregnancy and birth outcomes. Pregnant women with an active ED are at increased risk of experiencing antepartum hemorrhage [10], hyperemesis gravidarum [11], higher rates of miscarriage [12], caesarean sections, and postpartum depression [13]. The literature on fetal outcomes of women with EDs displays lower [14] and higher birth weights [15,16], intrauterine growth restriction [10], small head circumferences [17], neurobehavioral dysregulations early after birth [18] as well as premature deliveries and perinatal mortality [15]. Disturbances and dysfunctions related to nutrition and eating behaviors which are core symptoms of EDs might contribute to these adverse pregnancy outcomes seen in women with EDs. On the other hand, given that affected women are often unduly preoccupied by eating and weight-control practices, they might have a higher nutritional knowledge, especially in terms of nutrient sources [19]. Therefore, one could also assume that at least subgroups of women with EDs might have a higher diet quality during pregnancy. Although most studies report an improvement in the ED symptomatology during pregnancy [20,21,22,23], there is limited knowledge about the actual food intake and overall dietary behavior of pregnant women with EDs.

The present systematic review aims to provide a synthesis of evidence regarding overall nutrition and related issues of pregnant women with a history of EDs. The main research questions are:

1. Do pregnant women with a history of EDs show different dietary intakes and patterns as compared to healthy pregnant women?
2. Do pregnant women with a history of EDs deviate from international dietary recommendations guidelines for pregnancy?
3. Do pregnant women with a history of EDs differ from healthy pregnant women with regard to nutritional biomarkers and dietary supplement intake?
4. Do pregnant women with a history of EDs show dysfunctional eating behaviors, i.e., restrictive eating, dieting, binge eating? Here, we will focus exclusively on behavioral *eating* aspects (which also represent ED symptoms), but not solely on ED symptoms with no direct connection to food intake (e.g., self-induced vomiting).

## 2. Methods

The review process was conducted according to the ‘Preferred Reporting Items for Systematic Reviews and Meta-Analyses’ (PRISMA) [24]. Papers were searched on PubMed and PsycInfo and covered a period ranging from 1987 to November 2018. The approach of this systematic review was specified in advance and documented in a protocol.

### 2.1. Search Strategy

Studies were identified by searching electronic databases and scanning reference lists of articles for relevant studies. The search was applied using the scientific databases PubMed and PsycInfo. In addition, we handsearched contents pages of ‘The International Journal of Eating Disorders’, the ‘Journal of Eating Disorders’, the ‘European Eating Disorders Review’, ‘Eating Disorders: The Journal of Treatment and Prevention’ and ‘Nutrients’.

Until November 2018, a search of two databases was performed using the terms ‘nutrition’, ‘food intake’, ‘eating behavior’, ‘pregnancy’, and ‘eating disorders’. As an example, for the PubMed search, the search term was defined as follows:

‘((((“nutritional status”[MeSH Terms] OR (“nutritional”[All Fields] AND “status”[All Fields]) OR “nutritional status”[All Fields] OR “nutrition”[All Fields] OR “nutritional sciences”[MeSH Terms] OR (“nutritional”[All Fields] AND “sciences”[All Fields]) OR “nutritional sciences”[All Fields]) OR (“eating”[MeSH Terms] OR “eating”[All Fields] OR (“food”[All Fields] AND “intake”[All Fields]) OR “food intake”[All Fields])) OR (“eating behaviour”[All Fields] OR “feeding behavior”[MeSH Terms] OR (“feeding”[All Fields] AND “behavior”[All Fields]) OR “feeding behavior”[All Fields] OR (“eating”[All Fields] AND “behavior”[All Fields]) OR “eating behavior”[All Fields])) AND (“pregnancy”[MeSH Terms] OR “pregnancy”[All Fields])) AND (“feeding and eating disorders”[MeSH Terms] OR (“feeding”[All Fields] AND “eating”[All Fields] AND “disorders”[All Fields]) OR “feeding and eating disorders”[All Fields] OR (“eating”[All Fields] AND “disorders”[All Fields]) OR “eating disorders”[All Fields]) AND “humans”[MeSH Terms]’.

The first stage of study selection included the removal of duplicate studies, conducted by the paper’s first author (A.F.D.). The first and the fourth author (S.B.L.) roughly screened the remaining studies by scanning article titles and abstracts. In the next step, the same two authors performed full text examinations of potentially relevant studies according to the defined eligibility criteria. Disagreements were resolved by discussion between the two investigators; if no agreement could be reached, the last review author (K.E.G.) was consulted.

The first author then extracted information from each included study using a specifically designed data collection sheet. The data collection sheet sought the following variables: (i) study characteristics (including study design, follow-up period, study size, country, funding sources), and the trial’s inclusion and exclusion criteria; (ii) characteristics of trial participants (including method of ED diagnosis, gestational week); (iii) type of outcome measures (including dietary quantity and quality, eating patterns, dietary supplement use, nutritional biomarkers, and eating behavior in general), and results (including limitations, strengths, clinical implications and conclusions).

### 2.2. Eligibility Criteria

Eligibility criteria were based on the five PICOS dimensions, i.e., participants (P), interventions (I), comparators (C), outcome (O) and study design (S) [25].

#### 2.2.1. Participants

Participants included pregnant women of any age with a past or current ED. Eating disorders were defined as AN, BN, BED and OSFED, previously known as ‘Eating Disorder Not Otherwise Specified’ (EDNOS), according to DSM criteria. Pica, an eating disorder that involves the persistent eating of substances that are not food and do not provide nutritional value, was defined as exclusion criterion for this review, as Pica is often described inaccurately as a part of culturally supported or socially normative practices [26]. Pregnant women with additional severe mental disorders (e.g., dementia, schizophrenia) as well as somatic syndromes influencing weight or eating behavior (e.g., diabetes mellitus, epilepsy, Prader-Willi syndrome), or virus infections (e.g., malaria) were excluded from this review.

#### 2.2.2. Interventions

Trials assessing the dietary intake and eating patterns of pregnant women with a history of EDs in comparison to pregnant women without EDs. Dietary intake includes dietary quality, dietary supplement use, and eating behavior in general. Additionally, this review includes studies reporting on nutritional biomarkers of under- or malnutrition.

#### 2.2.3. Comparators

Studies were eligible if a control group was included that consisted of pregnant women without any EDs. If there was no control group assessed, international dietary recommendation guidelines for pregnancy were used as a comparator [1,27,28].

#### 2.2.4. Outcome

*Primary outcome measures:* Dietary intake in terms of food quantity and food quality, eating patterns regarding different forms of nutrition (e.g., vegetarianism, veganism), dietary supplement use (e.g., vitamin supplements), and nutritional biomarkers (e.g., ferritin). *Secondary outcome measures:* eating behavior in general (e.g., dieting or binge eating).

#### 2.2.5. Study design

Cross-sectional and longitudinal observational studies assessing the dietary intake and eating patterns of pregnant women with a history of EDs. No publication date restrictions were imposed. Unpublished material and abstracts were included; publications in form of book chapters, reviews, case reports/series or dissertations were excluded. This review was limited to studies in English, French or German language.

## 3. Results

**Figure 1:**
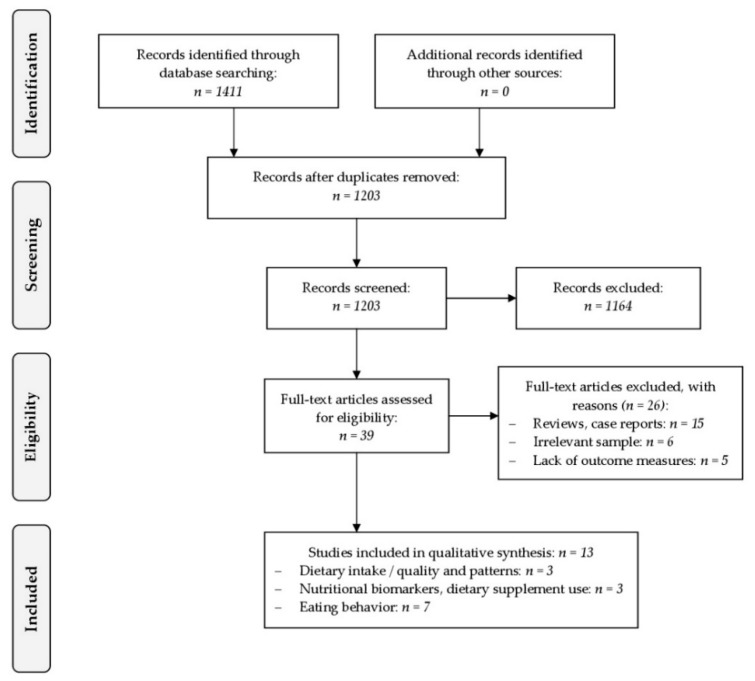
PRISMA flow chart for study inclusion.

A total of 13 studies were identified for inclusion in this review. Interrater reliability was good, with κ = 0.71. See the flow diagram for an overview of the study selection process (Figure 1).

We clustered the 13 studies according to the following topics concerning pregnant women with EDs:

1. Dietary intake/quality and patterns (*n* = 3)
2. Nutritional biomarkers and dietary supplement use (*n* = 3)
3. Eating behavior (*n* = 7)

### 3.1. Studies Investigating Dietary Intake and Patterns in Pregnant Women with Eating Disorders

We identified three studies which investigated dietary intakes in pregnancy in women with lifetime EDs [29,30,31]. All three studies were cross-sectional analyses, using data from large population-based cohort studies (see Table 1). Women with lifetime EDs were compared with control women free of any EDs before or during pregnancy. Maternal history of lifetime EDs was assessed during pregnancy [31] with self-reported questionnaires, either at gestational week (GW) 12 [29], or at six months prior to pregnancy and at GW 18.1 [30]. Dietary information was collected via food frequency questionnaires (FFQs) in the first half of the pregnancy [30,31] and in the third trimester [29]. The FFQ determines the frequency of consumption and the portion size of a wide variety of foods and drinks. Additionally, Micali et al. [29] and Siega-Riz et al. [30] combined related foods into different food groups (e.g., red meat, poultry, sausages/burgers, pies/pasties = ‘meat’). Two of the three studies [29,30] examined the exact dietary intake of pregnant women across the ED spectrum in comparison to pregnant women without EDs. Siega-Riz et al. [30] focused on nutrient and food group intakes of pregnant women with BED and BN in the second trimester. Micali et al. [29] examined the dietary intake as well as dietary patterns of pregnant women with AN, BN or both (AN + BN) in the third trimester.

In terms of macronutrient intake, there were no differences to healthy pregnant women in energy, carbohydrate, fat and protein consumption of pregnant women with AN, BN or both in the third trimester [29]. In contrast, women with BED before and during pregnancy showed a higher consumption of total energy and total fat (monounsaturated fatty acids, MUFA; saturated fatty acids, SFA) in the second trimester [30]. Women who developed BED during pregnancy also had a higher total energy and SFA intake [30].

With regard to micronutrient intake through diet, there was very little difference in pregnancy in women with EDs compared with control women [29,30]. In particular, women in the second trimester with BED before and during pregnancy had lower intakes of folate, potassium and vitamin C [30]. This is reflected in the food group consumption, showing lower intakes of fruit and juices in those women [30]. In the third trimester, women with AN and AN + BN had higher folate and potassium intakes; women with AN + BN showed higher intakes of calcium, phosphorus, zinc, and vitamin C and women with BN had a slightly higher vitamin E intake compared to control women [29]. In general, the average mineral and vitamin intakes were satisfying in women with EDs throughout pregnancy [27,28].

In relation to food group consumption, women with BED before and during pregnancy had higher intakes of overall fat (butter, margarines, and oils) and milk desserts in the second trimester [30]. Pregnant women with AN, BN or both in the third trimester were less likely to use butter and drink full-fat milk in favor of skimmed and soya milk [29]. Those women also consumed less meat and fewer potatoes; however, they had a higher intake of soya products and pulses compared to the referent group [29]. Similarly, women with BN before and during pregnancy had a lower intake of high-fat meats in the second trimester [30]. Moreover, a higher consumption of artificially sweetened beverages among women with active and past BN and BED was observed [30]. Women with an active BED showed a slight increase in mean coffee consumption and women with lifetime AN and AN + BN reported a significantly higher coffee consumption (>355 mg caffeine/week) during pregnancy [29,30].

Regarding dietary patterns, women with lifetime EDs scored higher on the vegetarian dietary pattern (high intakes of meat substitutes, pulses, nuts and herbal teas, and high negative intakes of red meat and poultry), which corresponds to their self-report as being vegetarian [29]. Women with a lifetime AN scored higher on the ‘traditional’ dietary pattern (high loadings for all types of vegetables and red meat and poultry); women with a lifetime AN + BN scored higher on the ‘health conscious’ (high intake of salad, fruit, rice, pasta, oat and bran-based breakfast cereals, fish, pulses, fruit juices and non-white bread) and the ‘traditional’ dietary pattern [29].

Nguyen et al. [31] calculated a diet quality score with the data of the FFQs on the basis of the Dutch national dietary guidelines, including 15 components and cut-offs (e.g., vegetables (≥200 g/day), dairy (≥300 g/day), and red meat (≤375 g/week)). After adjustment for socioeconomic and lifestyle factors (maternal age, ethnicity, educational level, BMI, household income, psychiatric symptoms), women with a history of EDs had a significantly higher diet quality score than women unexposed [31].

### 3.2. Studies Investigating Maternal Biomarkers of Nutrition and Dietary Supplement Use in Pregnant Women with Eating Disorders

We identified two studies which assessed maternal biomarkers of nutrition in pregnant women with a history of EDs [15,32], and one study which focused on the dietary supplement use in those women [33] (see Table 2).

Koubaa et al. [32] examined biomarkers of nutrition and stress during early pregnancy in a cohort of women with a previous history of AN or BN in comparison to healthy women. Women with a history of AN showed a significantly higher frequency of anemia (hemoglobin, Hb < 110 g/L), which was related to low levels of maternal serum ferritin in early pregnancy compared to controls (*p* < 0.01). In their large register search study (*n* = 4299), Linna et al. [15] also found an increased occurrence of anemia in women with AN compared to healthy controls.

Regarding dietary supplement use, Dellava et al. [33] performed a cross-sectional analysis of the Norwegian Mother and Child Cohort Study (MoBa). Over 90% of women with an ED diagnosis as well as women without EDs used dietary supplements during pregnancy. Folic acid supplementation was the most common dietary supplement. After adjusting for covariates (maternal age, parity, smoking, household income, and educational level), no significant differences existed across groups for the use of any dietary supplement during pregnancy. However, women with EDNOS-P (purging subtype) were significantly more likely to take iron supplements during pregnancy (*p* < 0.04) [33].

### 3.3. Studies Investigating Eating Behavior in Pregnant Women with EDs

Concerning dysfunctional eating behaviors in pregnant women with a history of EDs, we identified seven relevant studies [20,23,34,35,36,37,38]. These studies provide evidence for a general improvement in dysfunctional eating behaviors during pregnancy in women with a recent or past ED (see Table 3). Women who actively suffered from BN during pregnancy reported an improvement of objective binge episodes with each passing trimester [36,37]. In particular, the frequency of objective binge eating in pregnant women with full or partial AN, BN or BED decreased to 2.9 episodes per 28 days intrapartum compared to 8.7 episodes prepartum [23]. 65% of pregnant women with an active BN, AN or mixed symptoms (AN + BN) who restricted their intake reported that they improved nutritionally or stopped restricting entirely; 56% of those who binged improved during pregnancy [38]. Blais et al. [20] also found a decrease in binge frequency in women with an active BN intrapartum, but no significant differences for binge frequencies and restrictive eating were seen in women with an active AN. For pregnant women with an active AN, BN or both, there was also evidence that bingeing became worse in pregnancy (18%) and that increased impulsivity within food intake resulted in overeating in 12% of those women [38]. Cross-sectional analyses of the Avon Longitudinal Study of Parents and Children (ALSPAC) revealed that 11.3% of women with a recent ED and 4.4% of women with a past ED dieted in late pregnancy, compared to 8% of obese and 2.5% of nonobese control women [34]. Regarding the question of whether they felt a loss of control over eating (LOC) during pregnancy, 72.5% of women with a recent ED and 42.8% of women with a past ED as well as 33.8% of healthy obese women and 36.1% of healthy nonobese women confirmed this question. Especially women with a recent BN were more likely to report LOC in pregnancy compared to all other groups [34].

## 4. Discussion

This systematic review analyzed diet quality, nutritional biomarkers and dietary supplement use as well as dysfunctional eating behaviors in women with a current or past ED during pregnancy. Given the fact that, up to now, only three studies have investigated dietary intake and diet quality in women with EDs during pregnancy [29,30,31], and only one study has investigated nutritional biomarkers [32] and dietary supplement use [33] in pregnant women with EDs, a clear finding of our review is that more research in this area is urgently needed.

### 4.1. Dietary Intake and Patterns in Pregnant Women with Eating Disorders

Summarizing the current findings of the three relevant studies, there is congruent evidence for an adequate diet in pregnant women with EDs, although there are specific subgroup differences [29,30,31].

The strengths of these studies are their population-based, longitudinal design, and therefore very large sample sizes. However, all cited studies have a general limitation regarding the examination of food intake with the use of food frequency questionnaires, as they rely on memory and reported intakes, which are subject to measurement errors [39]. Moreover, the items of the FFQ are adapted on the specific diet of a country; thus, carefulness is required regarding participants with different ethnic backgrounds [31]. Another weakness is the use of standard portion sizes to assess nutrient intakes from the FFQs, resulting in imprecise estimates of the actual food quantity [29,30]. It remains unclear whether the FFQ is a valid tool for the dietary assessment in pregnant women with EDs who experience worries and feelings of guilt or embarrassment for not caring for their unborn children, as they may under- or over-report specific food items (e.g., energy-dense foods) [31]. However, several studies conclude that the FFQ is a valid tool for ranking women in accordance with their nutrient intakes during pregnancy [40,41,42].

Nguyen et al. calculated a higher diet quality score for women with a history of EDs, not differentiating the disorder into active, past or type of ED [31]. Furthermore, the exact dietary data was not presented in the article. Micali et al. examined nutrient and food group intakes of pregnant women with lifetime AN, BN and both [29]; Siega-Riz et al. investigated those of women with active BN and active BED [30]. Consequently, except for the BN group, the other subgroups cannot be compared on the basis of uniform diagnoses between those studies. Nevertheless, the data on dietary intake in pregnant women with a history of AN, BN and AN + BN suggests a relatively good diet quality, supported by a vegetarian dietary pattern with lower intakes of meat and similar protein, fat and carbohydrate intakes compared to controls. However, these results can also be the result of persistent ED symptoms during pregnancy [31]. More precisely, women whose ED cognitions are present in pregnancy might have made more informed choices about a ‘healthy’ diet that included low caloric foods (e.g., vegetables, skimmed milk) to prevent further weight gain [31] or to avoid ‘fattening’ foods during an interval when purging episodes may be reduced [30]. If so, those women would have scored higher on the diet quality. An alternative explanation for better nutrition in women with a history of AN, BN or both is an improvement in the ED symptoms during pregnancy, which is suggested by the majority of previous studies in this field [20,21,22,23]. This is supported by the notion that, especially during pregnancy, expectant mothers are often highly motivated to optimize their lifestyle [1]. However, women with BED before and during pregnancy showed higher energy and overall fat intakes and lower intakes of some nutrients compared to controls. For women with BED, pregnancy could be a trigger for the continuation or even deterioration of symptoms [22].

The higher consumption of artificially sweetened beverages among women with active and past BN and BED [24] might be a reflection of their ED pattern in reducing added sugars. Summarized data from three very large prospective cohort studies showed several risks which were associated with the consumption of artificially sweetened beverages by pregnant women, including prematurity and the diagnosis of asthma in their children up to the age of seven years [43].

Similarly, the high consumption of caffeine (>355 mg/day), especially in women with AN and AN + BN, is of high concern, since caffeine and coffee consumption during pregnancy might be associated with spontaneous abortion at daily levels of 300 mg caffeine and above in a dose-dependent way (7–19% for every increase in caffeine intake of 100–150 mg/day) [44,45]. Based on limited data, a Cochrane meta-analysis was unable to conclude on the effectiveness of the abstention from caffeine on birth weight or other relevant endpoints [46]. In a clinical sample of ED patients, the main reasons for consuming caffeinated beverages were appetite suppression, feeling of fullness, facilitation with purging, and increasing the metabolic rate to enhance energy expenditure [47]. There is evidence that habitual coffee consumption plays a possible role in weight loss (increased metabolic rate, energy expenditure, lipid oxidation, and lipolytic and thermogenic activities) [48]. However, the American College of Obstetricians (ACOG) and the European Food Safety Authority (EFSA) recommend that pregnant women should drink no more than 200 mg of caffeine per day [49,50].

Lastly, the average intake of all nutrients was sufficient for women with active and past EDs, except for potassium and phosphorus being very high and iron and folate being much lower than recommended in the analyses by Siega-Riz et al. [30]. However, nutrient intakes from supplement use were not included in the analyses.

### 4.2. Maternal Biomarkers of Nutrition and Dietary Supplement Use in Pregnant Women with Eating Disorders

Both studies that identified nutritional biomarkers during pregnancy showed an increased risk of maternal iron deficiency anemia (IDA) for women with AN during early pregnancy [15,32]. However, data of the nutritional biomarkers throughout pregnancy was not available, since the determination was performed only in one blood sample in early pregnancy [32]. The etiology of anemia is multifactorial and includes insufficient dietary intake of folate, vitamin B_12_ and A, along with iron, which is the most common nutritional cause [51]. According to previous World Health Organization (WHO) reports, on average, about 50% of all cases of anemia in pregnancy were due to iron deficiency [52]. During pregnancy, there is an increased need for iron, because more iron is required for the fetus, placenta and 20% increased erythrocytes in the expectant mother [1]. Iron deficiency in pregnancy increases the risk of premature birth, low birth weight and irreversible or partially reversible neurobehavioral and cognitive impairments [32,51,53,54,55]. For instance, Koubaa et al. found that maternal serum levels of ferritin in women with a history of AN correlated strongly with impaired memory function in their children at five years of age [32]. Linna et al. did not relate maternal iron status with newborn outcomes, but they reported that maternal AN was associated with several adverse perinatal outcomes (e.g., slow fetal growth, very premature birth, small for gestational age, low birth weight), which could be mediated by low folate or iron intakes during pregnancy [15]. If an IDA is medically diagnosed, iron supplementation in addition to sufficient intake of iron-rich foods is recommended [1]. Therefore, pregnant women with a history of EDs, and particularly of AN, should be closely monitored in terms of nutrient deficiencies.

To our knowledge, the study by Dellava et al. is the only study to date that examined the dietary supplement intake in pregnant women with EDs compared to control women [26]. Unfortunately, it was not possible to determine if women with EDs consumed more or less micronutrients from dietary supplements than women without EDs, since information of the amount of each item contained in the dietary supplements was not collected [26]. It would also be important to know whether women with EDs were instructed by their gynecologist to take dietary supplements [26]. Over 90% of women with EDs used dietary supplements during pregnancy, with folic acid being the most commonly used supplement. Folic acid supplement use was higher during pregnancy than periconceptional for all women [26]. Numerous epidemiological studies and subsequent meta-analyses have shown that a periconceptional folic acid supplementation of 400 μg (alone or in combination with other micronutrients) can reduce the risk of childhood malformations of the nervous system (neural tube defects) [56,57]. However, in the study by Dellava et al., the increase in folic acid supplementation prior to pregnancy in women with and without EDs was likely too late to prevent birth defects associated with folate deficiency according to the Norwegian recommendations [26]. Therefore, involved specialists should motivate women with EDs to supplement critical nutrients (e.g., folate, iron, iodine). Supplements could easily be accepted by these women, as they are not associated with caloric intake [26]. However, in women with purging behavior, it is questionable as to how many supplemented nutrients might be absorbed at the intestinal absorption site.

### 4.3. Dysfunctional Eating Behavior in Pregnant Women with EDs

In terms of behavioral aspects of eating, pregnancy is associated with an overall improvement in the severity of dysfunctional eating behavior for most women [20,23,34,35,36,37,38]. Reasons for the improvement in eating behavior may include the relief of a sense of responsibility for body weight and shape and the woman’s worries about its harming effects on her unborn child [37]. However, pregnancy might also be a trigger for the deterioration of bingeing and overeating [22,38]. After all, every tenth pregnant woman with a recent ED reports restrictive intake, which is worrisome [34]. Reasons for the intensification of dysfunctional eating behaviors may include increased anxiety over weight gain [38]. Furthermore, physiological changes in the course of pregnancy, such as changes in satiety associated with an altered leptin level, may have important influences on eating behavior [58]. The increased rates of LOC, both in women with and without EDs, might be correlated with general ravenous appetite experienced in pregnancy. Interestingly, in the study by Blais et al., pregnancy outcomes (lower live birth rates, higher therapeutic abortion rates) of women with AN and BN were not associated with the maternal age, or the presence of ED symptoms at conception [20].

Additionally, in the postpartum phase, the improvement seems to decline, and dysfunctional eating behaviors tend to return toward prepregnancy levels [29]. Therefore, pregnant women with eating disorders need enhanced intrapartum *and* postnatal support. From a nutritional point of view, the latter is of great importance regarding (breast)feeding practices in women with EDs. For example, in a study with 20 bulimic women, three mothers reported that they restricted the dietary intake of their children within the first year of life [36].

### 4.4. Strengths and Limitations

This is the first systematic review that has examined pregnancy nutrition and related issues in women with a history of EDs. The strengths of this review are its methodical procedure according to the PRISMA statement and the focus on nutritional aspects of eating disorders during pregnancy. Thus, this review draws attention to important gaps in the eating disorder research.

Given the fact that the studies eligible for this review were very heterogeneous, a quality rating and meta-analysis could not be performed. The articles were ranging over a time span of 30 years; consequently, consistent diagnostic criteria for EDs were not applied. Another major issue is the inadequate diagnostic identification of EDs through self-report in some of the reviewed studies. Therefore, a reporter bias and misclassification of EDs cannot be ruled out. Furthermore, women who reported an ED might have a milder form of ED, since women who suffer from a severe ED might not participate in research studies or might not report their ED accordingly due to potential feelings of sorrow and guilt. Consequently, the prevalence of women with ED and differences in dietary intake might be underestimated. Moreover, it was not possible to ascertain between active and lifetime ED during pregnancy in some studies, resulting in mixed study samples of reduced comparability. There is a limited generalizability in studies that extracted data from the MoBa study, since differences exist between the study sample and the general population in terms of educational level and multivitamin consumption. From the seven studies investigating dysfunctional eating behavior of pregnant women with EDs, three studies had small sample sizes [23,36,38], two studies relied on retrospective recall [35,37] and, unfortunately, only one study included a control group of women without EDs [34].

## 5. Conclusions and Future Directions

Based on our four research questions investigated in the present systematic review, our main findings are as follows: (1) considering the drawback that dietary intakes were assessed by self-report, pregnant women with a history of EDs show a similar dietary intake to healthy pregnant women; there is evidence that women with EDs have a sufficient diet quality and are more likely to follow a vegetarian dietary pattern. Women with BED seem to be an exception as they show inadequate energy and fat intakes; (2) the startling caffeine and coffee consumption, especially in pregnant women with AN and AN + BN, and the use of artificially sweetened beverages in pregnant women with active and past BN and BED is of high concern and needs further investigation in relation to fetal outcomes; (3) women with AN were at increased risk of experiencing iron deficiency anemia in pregnancy; anyhow, women with EDs did not differ in overall dietary supplement use compared to healthy women; (4) dysfunctional eating behaviors (bingeing, dieting) improved substantially with pregnancy.

We feel that the issue of EDs in pregnancy is still an understudied field. There is still no consistent answer to the underlying biological mechanisms that might explain the increased risk for adverse perinatal outcomes in women with EDs. As outlined above, most of the current evidence in the field is derived from self-report data, which is prone to biases, e.g., due to influences of social desirability or memory gaps. This highlights the need for method combinations, integrating data from self-report, observational data, and in order to elucidate consequences of nutrition practices for fetal development, ideally also from neuropsychological and neurobiological methods [32,59]. For example, fetal magnetoencephalography (fMEG) is a non-invasive method to study fetal brain activity, which has previously revealed evidence that the maternal metabolism might program the fetal brain [60,61]. However, the first step is the adequate assessment and identification of EDs through medical professionals (general practitioner, gynecologist, or fertility specialist), ideally before pregnancy [62]. Paslakis and de Zwaan [62] recently provided clinical recommendations and specific algorithms for the management of pregnant females with EDs and for females with EDs seeking fertility treatment, underlining the importance of an interdisciplinary approach.

Regarding the research gaps concerning pregnant women with EDs, nutrition quality/food intake (including the adherence to a vegan diet), gestational weight gain, gestational diabetes, and dysfunctional behaviors (including substance misuse) are just a few topics which urgently require further investigation. Especially during pregnancy, women with EDs might be highly motivated to change their behavior in favor of their developing child. Therefore, studies on the treatment of EDs during pregnancy are urgently needed to ensure an optimal development of the fetus and to contribute to the prevention of intergenerational transmission of EDs.

Based on the evidence available, the assessment of maternal nutritional status is complex, but especially needed for vulnerable groups like women with EDs during pregnancy. With the exception of women with BED, food intake seems sufficient during pregnancy and dysfunctional eating habits are improved, which is an encouraging finding. Particularly women with active EDs might need nutritional counselling and close monitoring of nutrient deficits. More research is needed to confirm previous studies and to identify treatment interventions that support lasting improvements in eating behaviors and dietary intake in the postpartum phase.

## Figures and Tables

**Table 1:** Studies investigating dietary intake/quality/patterns in pregnant women with eating disorders.

Source	Study Design	Country	Sample	ED Diagnosis	*n*	Prevalence of EDs	Outcome	Dietary Information
Nguyen et al., 2017 [31]	Cross-sectional analysis of the Generation R study	Netherlands	Pregnant women with and without a history of any ED	Self-reported questionnaire and clinical diagnoses (subsample; *n* = 928) during pregnancy	6196	9.5% (*n* = 591)	Diet quality score, including 15 components and cut-offs (e.g., vegetables (≥200 g/day), dairy (≥300 g/day), red meat (≤375 g/week))	Semi-quantitative modified 293-item FFQ at 13.6 weeks of gestation (12.4–16.2)
**Main findings**	Women with a history of EDs had a higher diet quality score than women without EDs							
Micali et al., 2012 [29]	Cross-sectional analysis of the ALSPAC study	United Kingdom	Pregnant women with and without a lifetime AN, BN, and AN + BN (exclusion: non-singleton pregnancies, miscarriage)	Self-reported questionnaire at 12 weeks of gestation	10,137	4.1% (*n* = 414); AN (*n* = 151); BN (*n* = 186); AN + BN (*n* = 77)	Frequency of consumption of various food groups, daily nutrient intakes (macronutrients), and dietary patterns	FFQ at 32 weeks of gestation; 14 food groups, 5 dietary patterns
**Main findings** (compared to control women)	1. Women with lifetime ED: - 2.8 times more likely to describe themselves as vegetarian - ↑ scoring on ‘vegetarian’ dietary pattern - ↓ meat, potatoes, SFA (AN, AN + BN) - ↑ soya products, pulses, skimmed and soya milk, fiber - ↓ butter, full-fat milk - ↑ caffeine (>355 mg/day) (esp. AN, AN + BN)					2. Women with a lifetime AN: - ↑ scoring on ‘traditional’ dietary pattern 3. Women with a lifetime BN: - ↑ bread (1.1 slices/d more), PUFA - ↓ sugar, non-milk extrinsic sugar 4. Women with a lifetime AN + BN: - ↑ scoring on ‘health conscious’ and ‘traditional’ dietary pattern		
Siega-Riz et al., 2008 [30]	Cross-sectional analysis of the MoBA study	Norway	Pregnant women with and without lifetime BN and BED	Self-reported questionnaire 6 month before and during pregnancy (18.1 week of gestation)	30,040	6.1% lifetime ED (*n* = 1840); 4.6% active ED (*n* = 1393); BN/BN (*n* = 59); BN/BED (*n* = 60); BED/BED (*n* = 650); none/BED (*n* = 624)	Nutrient and food group intakes	Semi-quantitative FFQ at gestational weeks 15–22; 255 questions, 20 food groups
**Main findings** (compared to control women)	1. Women with BED before + during pregnancy: - ↑ total energy (2459.0 ± 30.2 kcal vs. 2348.3 ± 4.6 kcal) - ↑ total fat (87.5 ± 1.2 g vs. 80.8 ± 0.2 g) - ↑ MUFA (27.8 ± 0.4 g vs. 26.6 ± 0.1 g) - ↑ SFA (34.2 ± 0.5 g vs. 31.3 ± 0.1 g) - ↓ folate (268.9 ± 3.8 µg vs. 273.4 ± 0.7 µg) - ↓ potassium (4008.1 ± 49 mg vs. 4018.6 ± 7.6 mg) - ↓ vitamin C (155.5 ± 3.7 mg vs. 167.5 ± 0.6 mg) - ↑ butter, margarines, oils, milk desserts, candy, coffee - ↓ fruit, juices, chicken					2. Women with incident BED during pregnancy: - ↑ total energy (2544.1 ± 41.2 kcal vs. 2348.3 ± 4.6 kcal) - ↑ SFA (34.9 ± 0.6 g vs. 31.3 ± 0.1 g) - ↑ cakes, candy, milk desserts, coffee; ↓ juices 3. Women with BN before and during pregnancy: - ↑ yogurt, cheeses - ↓ sweetened beverages, high-fat meats		

Abbreviations: ED: eating disorder; AN: Anorexia Nervosa; BN: Bulimia Nervosa; BED: Binge Eating Disorder; ALSPAC: Avon Longitudinal Study of Parents and Children: MoBa: Norwegian Mother and Child Cohort Study; FFQ: Food Frequency Questionnaire; ↑: higher (consumption); ↓: lower (consumption); SFA: saturated fatty acids; MUFA: monounsaturated fatty acids; PUFA: polyunsaturated fatty acids; BN/BN = BN before and during pregnancy; BN/BED = BN before, BED during pregnancy; BED/BED = BED before and during pregnancy; none/BED = no ED before, BED during pregnancy.

**Table 2:** Studies investigating nutritional biomarkers and dietary supplement use in pregnant women with eating disorders.

Source	Study Design	Country	Sample	ED Diagnosis	*n*	Prevalence of EDs	Outcome	Main Findings
Koubaa et al., 2015 [32]	Longitudinal cohort study (follow-up period: 1 year)	Sweden	Pregnant, nulliparous non-smoking women with and without a history of AN and BN	Interview according to DSM-IV diagnostic criteria; medical records	96	38.5% (*n* = 37); AN (active AN: *n* = 8; past AN: *n* = 12); BN (active BN: *n* = 1; past BN: *n* = 16)	Maternal serum biomarkers of nutrition and stress at 10 weeks of gestation within a routine blood sample (ferritin, cortisol, TSH, T4, insulin, IGF-I and IGFBP1)	Women with previous AN: - ↑ frequency (70%) of anemia (Hb < 110 g/L) compared to controls (12%) ↓ serum ferritin levels compared to controls
Linna et al., 2014 [15]	Register search study	Finland	Female ED patients and unexposed controls	Attending physicians at the clinic with ICD-10 (AN, BN, atypical AN/BN) and DSM-IV (BED) criteria	4299	15.3% (*n* = 657); AN (*n* = 182); BN (*n* = 436); BED (*n* = 39)	Pregnancy complications (obtained from Medical Birth Register): gestational diabetes mellitus, initiation of insulin treatment, anemia, antenatal corticosteroid treatment, pregnancy-related ICD-10 diagnoses	Anemia was more frequent among women with AN (3.97%) compared with unexposed women (1.54%)
Dellava et al., 2013 [33]	Cross-sectional analysis of the MoBA study	Norway	Pregnant women across eating disorder subtypes compared with a referent group	Self-reported questionnaire at GW 19	37,307	6.3% (*n* = 2348); AN (*n* = 34); BN (*n* = 326); BED (*n* = 1944); EDNOS-P (*n* = 44)	Use of dietary supplements (checklist including 22 specific nutrients, at three time points prior to pregnancy (≥9 weeks, 8–5 weeks and 4–1 week before conception) and eight time periods during pregnancy (GW 1–4, 5–8, 9–12 13–16, 17–20, 21–24, 25–28, and 29+)	Dietary supplement use during pregnancy was as follows (between group differences were not statistically significant): - 91.2% of women with AN - 92.2% of women with BN - 93.2% of women with EDNOS-P (↑ intake of Fe-containing supplements) - 90.6% of women with BED - 93.5% of the women without EDs

Abbreviations: ED: eating disorder; AN: Anorexia Nervosa; BN: Bulimia Nervosa; BED: Binge Eating Disorder; EDNOS-P: Eating Disorder Not Otherwise Specified – Purging subtype; MoBa: Norwegian Mother and Child Cohort Study; TSH: Thyroid-Stimulating Hormone; T4: Free Thyroxine (T4); IGF-I: Insulin-Like Growth Factor I; IGFBP1: IGF Binding Protein 1; Hb: Hemoglobin; Fe: Iron; DSM: Diagnostic and Statistical Manual of Mental Disorders; ICD-10: International Statistical Classification of Diseases and Related Health Problems; GW: gestational week; ↑: higher; ↓: lower.

**Table 3:** Studies investigating eating behavior in pregnant women with eating disorders.

Source	Study Design	Country	Sample	ED Diagnosis	*n*	Prevalence of EDs	Outcome	Main Findings
Crow et al., 2008 [23]	Cross-sectional analysis of the McKnight Longitudinal Study of Eating Disorders	United States of America	Pregnant women with full/subthreshold AN, BN or BED	EDE	42	AN (*n* = 5; 11.9%); BN (*n* = 15; 35.7%); BED (*n* = 4; 9.5%); partial AN (*n* = 5; 11.9%); partial BN (*n* = 10; 23.8%); partial BED (*n* = 3; 7.1%)	Eating behaviors and disordered eating cognitions over the course of pregnancy	Frequency of objective binge eating episodes per 28 days intrapartum: 2.9 (vs. 8.7 prepartum)
Micali et al., 2007 [34]	Cross-sectional analysis of the ALSPAC study	United Kingdom	Pregnant women with and without recent/past AN, BN, AN + BN	Self-reported questionnaire at 12 weeks of gestation	12,252	0.5% recent ED (*n* = 57; 6 AN, 51 BN); 3.2% past ED (*n* = 395; 167 AN, 158 BN, 70 AN + BN)	18 GW: Self-induced vomiting, laxative use, exercise behavior, shape and weight concern; 32 GW: appraisals about weight gain during pregnancy, dieting, LOC	Dieting in pregnancy: - recent ED: 11.3% - past ED: 4.4% - nonobese controls: 2.5% LOC: - recent ED: 72.5% - past ED: 42.8% - nonobese controls: 36.1% Women with recent BN were more likely to report LOC in pregnancy compared to all other groups
Crow et al., 2004 [35]	Cross-sectional analysis of a longitudinal study (follow-up period: 10 – 15 years)	United States of America	Pregnant women with BN	EDI; SCID; Eating Disorders Questionnaire; self-report of BN symptoms	129	all BN	Bulimic symptoms, alcohol, drug, and tobacco use during pregnancy	Frequency of binge eating during pregnancy was rated as: - decreased by 59.6% - increased by 7.4% - unchanged by 33.0% The prevalence of alcohol use decreased after pregnancy was recognized (35% vs. 14%)
Blais et al., 2000 [20]	Longitudinal Study	United States of America	Pregnant women with AN and BN	LIFE-EAT II every 6 month	82	31,7% AN (AN-R: *n* = 7; AN-BP: *n* = 19); 68.3% BN (*n* = 56)	Pregnancy outcome (live birth, therapeutic/spontaneous abortion), ED symptomatology (restrictive eating, binging, etc.)	BN subjects: ↓ frequency of binging from prepregnancy to post-pregnancy AN subjects: No significant differences were seen for binging frequency and restrictive eating
Morgan et al., 1999 [37]	Retrospective analysis	United Kingdom	Pregnant women actively suffering from BN	EDE, SCID	94	all BN	Symptoms of bulimia nervosa and associated psychopathology at conception, each trimester and postnatally	Objective binge episodes improved with each passing trimester of pregnancy
Lemberg et al., 1989 [38]	Longitudinal study	United States of America	Pregnant women with an active AN, BN or mixed symptoms	Retrospective, 55-item questionnaire on ED symptoms and course of pregnancy	43	57% AN-R; 77% BN; 16% combination of both	Eating behaviors both antenatal and postnatal	Women who restricted their intake (*n* = 36): - 65% reported improved nutrition or stopping restricting // complete cessation: 7% Women who binged (*n* = 41): - improvement: 56% // worsening: 18.6% // complete cessation: 14% All women: 12% ↑ impulsivity with food by overeating
Lacey et al., 1987 [36]	Longitudinal study	United Kingdom	Pregnant untreated BN women	St George’s Hospital eating disorder unit; DSM-III	20	all BN	Impact of pregnancy on dietary difficulties of the bulimic woman	19 of the 20 subjects reduced frequency of binge eating over the course of pregnancy; only 5 patients were binge eating during the 3rd trimester; 75% having a complete cessation of binging by the 3rd trimester

Abbreviations: ED: eating disorder; AN: Anorexia Nervosa; AN-R: Anorexia Nervosa Restrictive subtype; AN-BP: Anorexia Nervosa Binge-Purge subtype; BN: Bulimia Nervosa; BED: Binge Eating Disorder; LOC: Loss of control over eating; ALSPAC: Avon Longitudinal Study of Parents and Children; EDE: Eating Disorder Examination; Eating Disorder Inventory; Structured Clinical Interview for DSM Axis I Disorders (SCID); LIFE-EAT II: Eating Disorders Longitudinal Interval Follow-up Evaluation; DSM: Diagnostic and Statistical Manual of Mental Disorders; GW: gestational week; ↑: higher; ↓: lower.

## References

[1] Koletzko B., Cremer M., Flothkötter M., Graf C., Hauner H., Hellmers C., Kersting M., Krawinkel M., Przyrembel H., Röbl-Mathieu M. (2018). Diet and lifestyle before and during pregnancy—Practical recommendations of the germany-wide healthy start—Young family network. Geburtshilfe Frauenheilkd..

[2] Berti C., Biesalski H.K., Gärtner R., Lapillonne A., Pietrzik K., Poston L., Redman C., Koletzko B., Cetin I. (2011). Micronutrients in pregnancy: Current knowledge and unresolved questions. Clin. Nutr..

[3] Lucas A. (2005). Long-term programming effects of early nutrition—Implications for the preterm infant. J. Perinatol..

[4] Barker D.J. (1990). The fetal and infant origins of adult disease. BMJ (Clin. Res. Ed.).

[5] Hoffman D.J., Reynolds R.M., Hardy D.B. (2017). Developmental origins of health and disease: Current knowledge and potential mechanisms. Nutr. Rev..

[6] Barker D.J.P. (2007). The origins of the developmental origins theory. J. Intern. Med..

[7] Gluckman P.D., Hanson M.A., Cooper C., Thornburg K.L. (2008). Effect of in utero and early-life conditions on adult health and disease. New Engl. J. Med..

[8] Easter A., Bye A., Taborelli E., Corfield F., Schmidt U., Treasure J., Micali N. (2013). Recognising the symptoms: How common are eating disorders in pregnancy?. Eur. Eat. Disord. Rev..

[9] APA (2013). Diagnostic and Statistical Manual of Mental Disorders.

[10] Eagles J.M., Lee A.J., Raja E.A., Millar H.R., Bhattacharya S. (2012). Pregnancy outcomes of women with and without a history of anorexia nervosa. Psychol. Med..

[11] Koubaa S., Hallstrom T., Lindholm C., Hirschberg A.L. (2005). Pregnancy and neonatal outcomes in women with eating disorders. Obstet. Gynecol..

[12] Micali N., Simonoff E., Treasure J. (2007). Risk of major adverse perinatal outcomes in women with eating disorders. Br. J. Psychiatry.

[13] Franko D.L., Blais M.A., Becker A.E., Delinsky S.S., Greenwood D.N., Flores A.T., Ekeblad E.R., Eddy K.T., Herzog D.B. (2001). Pregnancy complications and neonatal outcomes in women with eating disorders. Am. J. Psychiatry.

[14] Solmi F., Sallis H., Stahl D., Treasure J., Micali N. (2014). Low birth weight in the offspring of women with anorexia nervosa. Epidemiol. Rev..

[15] Linna M.S., Raevuori A., Haukka J., Suvisaari J.M., Suokas J.T., Gissler M. (2014). Pregnancy, obstetric, and perinatal health outcomes in eating disorders. Am. J. Obstet. Gynecol..

[16] Bulik C.M., Von Holle A., Siega-Riz A.M., Torgersen L., Lie K.K., Hamer R.M., Berg C.K., Sullivan P., Reichborn-Kjennerud T. (2009). Birth outcomes in women with eating disorders in the norwegian mother and child cohort study (moba). Int. J. Eat. Disord..

[17] Koubaa S., Hallstrom T., Hagenas L., Hirschberg A.L. (2013). Retarded head growth and neurocognitive development in infants of mothers with a history of eating disorders: Longitudinal cohort study. BJOG Int. J. Obstet. Gynaecol..

[18] Barona M., Taborelli E., Corfield F., Pawlby S., Easter A., Schmidt U., Treasure J., Micali N. (2017). Neurobehavioural and cognitive development in infants born to mothers with eating disorders. J. Child Psychol. Psychiatry.

[19] HO A.S.L., SOH N.L., WALTER G., TOUYZ S. (2011). Comparison of nutrition knowledge among health professionals, patients with eating disorders and the general population. Nutr. Diet..

[20] Blais M.A., Becker A.E., Burwell R.A., Flores A.T., Nussbaum K.M., Greenwood D.N., Ekeblad E.R., Herzog D.B. (2000). Pregnancy: Outcome and impact on symptomatology in a cohort of eating-disordered women. Int. J. Eat. Disord..

[21] Micali N., Treasure J. (2009). Biological effects of a maternal ed on pregnancy and foetal development: A review. Eur. Eat. Disord. Rev..

[22] Bulik C.M., Von Holle A., Hamer R., Knoph Berg C., Torgersen L., Magnus P., Stoltenberg C., Siega-Riz A.M., Sullivan P., Reichborn-Kjennerud T. (2007). Patterns of remission, continuation and incidence of broadly defined eating disorders during early pregnancy in the norwegian mother and child cohort study (moba). Psychol. Med..

[23] Crow S.J., Agras W.S., Crosby R., Halmi K., Mitchell J.E. (2008). Eating disorder symptoms in pregnancy: A prospective study. Int. J. Eat. Disord..

[24] Moher D., Liberati A., Tetzlaff J., Altman D.G., The P.G. (2009). Preferred reporting items for systematic reviews and meta-analyses: The prisma statement. PLoS Med..

[25] Liberati A., Altman D.G., Tetzlaff J. (2009). The prisma statement for reporting systematic reviews and meta-analyses of studies that evaluate health care interventions: Explanation and elaboration. Ann. Intern. Med..

[26] NEDA PICA. https://www.nationaleatingdisorders.org/learn/by-eating-disorder/other/pica.

[27] Procter S.B., Campbell C.G. (2014). Position of the academy of nutrition and dietetics: Nutrition and lifestyle for a healthy pregnancy outcome. J. Acad. Nutr. Diet..

[28] Maternal and Child Nutrition. https://www.nice.org.uk/guidance/qs98/resources/maternal-and-child-nutrition-pdf-2098975759045.

[29] Micali N., Northstone K., Emmett P., Naumann U., Treasure J.L. (2012). Nutritional intake and dietary patterns in pregnancy: A longitudinal study of women with lifetime eating disorders. Br. J. Nutr..

[30] Siega-Riz A.M., Haugen M., Meltzer H.M., Von Holle A., Hamer R., Torgersen L., Knopf-Berg C., Reichborn-Kjennerud T., Bulik C.M. (2008). Nutrient and food group intakes of women with and without bulimia nervosa and binge eating disorder during pregnancy. Am. J. Clin. Nutr..

[31] Nguyen A.N., de Barse L.M., Tiemeier H., Jaddoe V.W.V., Franco O.H., Jansen P.W., Voortman T. (2017). Maternal history of eating disorders: Diet quality during pregnancy and infant feeding. Appetite.

[32] Koubaa S., Hallstrom T., Brismar K., Hellstrom P.M., Hirschberg A.L. (2015). Biomarkers of nutrition and stress in pregnant women with a history of eating disorders in relation to head circumference and neurocognitive function of the offspring. BMC Pregnancy Childbirth.

[33] Dellava J.E., Von Holle A., Torgersen L., Reichborn-Kjennerud T., Haugen M., Meltzer H.M., Bulik C.M. (2011). Dietary supplement use immediately before and during pregnancy in norwegian women with eating disorders. Int. J. Eat. Disord..

[34] Micali N., Treasure J., Simonoff E. (2007). Eating disorders symptoms in pregnancy: A longitudinal study of women with recent and past eating disorders and obesity. J. Psychosom. Res..

[35] Crow S.J., Keel P.K., Thuras P., Mitchell J.E. (2004). Bulimia symptoms and other risk behaviors during pregnancy in women with bulimia nervosa. Int. J. Eat. Disord..

[36] Lacey J.H., Smith G. (1987). Bulimia nervosa: The impact of pregnancy on mother and baby. Br. J. Psychiatry.

[37] Morgan J.F., Lacey J.H., Sedgwick P.M. (1999). Impact of pregnancy on bulimia nervosa. Br. J. Psychiatry.

[38] Lemberg R., Phillips J. (1989). The impact of pregnancy on anorexia nervosa and bulimia. Int. J. Eat. Disord..

[39] Rodrigo C.P., Aranceta J., Salvador G., Varela-Moreiras G. (2015). Food frequency questionnaires. Nutr. Hosp..

[40] Pinto E., Severo M., Correia S., Dos Santos Silva I., Lopes C., Barros H. (2010). Validity and reproducibility of a semi-quantitative food frequency questionnaire for use among portuguese pregnant women. Matern. Child Nutr..

[41] Brantsæter A.L., Haugen M., Alexander J., Meltzer H.M. (2008). Validity of a new food frequency questionnaire for pregnant women in the norwegian mother and child cohort study (moba). Matern. Child Nutr..

[42] McGowan C.A., Curran S., McAuliffe F.M. (2014). Relative validity of a food frequency questionnaire to assess nutrient intake in pregnant women. J. Hum. Nutr. Diet..

[43] Bernardo W., Simões R., Buzzini R., Nunes V., Glina F. (2016). Adverse effects of the consumption of artificial sweeteners—Systematic review. Rev. Da Assoc. Médica Bras..

[44] Li J., Zhao H., Song J.-M., Zhang J., Tang Y.-L., Xin C.-M. (2015). A meta-analysis of risk of pregnancy loss and caffeine and coffee consumption during pregnancy. Int. J. Gynecol. Obstet..

[45] Chen L.-W., Wu Y., Neelakantan N., Chong M.F.-F., Pan A., van Dam R.M. (2016). Maternal caffeine intake during pregnancy and risk of pregnancy loss: A categorical and dose–response meta-analysis of prospective studies. Public Health Nutr..

[46] Jahanfar S., Jaafar S.H. (2015). Effects of restricted caffeine intake by mother on fetal, neonatal and pregnancy outcomes. Cochrane Database Syst. Rev..

[47] Hart S., Abraham S., Franklin R.C., Russell J. (2011). The reasons why eating disorder patients drink. Eur. Eat. Disord. Rev..

[48] Cano-Marquina A., Tarín J.J., Cano A. (2013). The impact of coffee on health. Maturitas.

[49] EFSA NDA Panel (EFSA Panel on Dietetic Products, Nutrition and Allergies) (2015). Scientific opinion on the safety of caffeine. EFSA J..

[50] ACOG (2010). Committee opinion no. 462: Moderate caffeine consumption during pregnancy. Obstet. Gynecol..

[51] Scholl T.O. (2011). Maternal iron status: Relation to fetal growth, length of gestation, and iron endowment of the neonate. Nutr. Rev..

[52] McLean E., Cogswell M., Egli I., Wojdyla D., de Benoist B. (2009). Worldwide prevalence of anaemia, who vitamin and mineral nutrition information system, 1993–2005. Public Health Nutr..

[53] Milman N. (2012). Oral iron prophylaxis in pregnancy: Not too little and not too much!. J. Pregnancy.

[54] Menon K.C., Ferguson E.L., Thomson C.D., Gray A.R., Zodpey S., Saraf A., Das P.K., Skeaff S.A. (2016). Effects of anemia at different stages of gestation on infant outcomes. Nutrition.

[55] Scholl T.O., Hediger M.L., Fischer R.L., Shearer J.W. (1992). Anemia vs iron deficiency: Increased risk of preterm delivery in a prospective study. Am. J. Clin. Nutr..

[56] De-Regil L.M., Peña-Rosas J.P., Fernández-Gaxiola A.C., Rayco-Solon P. (2015). Effects and safety of periconceptional oral folate supplementation for preventing birth defects. Cochrane Database Syst. Rev..

[57] U.S. Preventive Services Task Force (2017). Folic acid supplementation for the prevention of neural tube defects: Us preventive services task force recommendation statement. JAMA.

[58] Henson M.C., Castracane V.D. (2006). Leptin in pregnancy: An update1. Biol. Reprod..

[59] Easter A., Taborelli E., Bye A., Zunszain P.A., Pariante C.M., Treasure J., Schmidt U., Micali N. (2017). Perinatal hypothalamic-pituitary-adrenal axis regulation among women with eating disorders and their infants. Psychoneuroendocrinology.

[60] Schleger F., Linder K., Walter L., Heni M., Brändle J., Brucker S., Pauluschke-Fröhlich J., Weiss M., Häring H.-U., Preissl H. (2018). Family history of diabetes is associated with delayed fetal postprandial brain activity. Front. Endocrinol..

[61] Fehlert E., Willmann K., Fritsche L., Linder K., Mat-Husin H., Schleger F., Weiss M., Kiefer-Schmidt I., Brucker S., Häring H.-U. (2017). Gestational diabetes alters the fetal heart rate variability during an oral glucose tolerance test: A fetal magnetocardiography study. BJOG Int. J. Obstet. Gynaecol..

[62] Paslakis G., de Zwaan M. (2019). Clinical management of females seeking fertility treatment and of pregnant females with eating disorders. Eur. Eat. Disord. Rev..

